# Editorial: Virulence of filamentous fungi and its interaction with plants

**DOI:** 10.3389/fcimb.2023.1168148

**Published:** 2023-03-31

**Authors:** Ziyi Yin, Xinyu Liu, Jie Huang, Yanjun Kou, Xinhua Ding

**Affiliations:** ^1^ State Key Laboratory of Crop Biology, Shandong Provincial Key Laboratory for Biology of Vegetable Diseases and Insect Pests, College of Plant Protection, Shandong Agricultural University, Taian, Shandong, China; ^2^ Department of Plant Pathology, College of Plant Protection, Nanjing Agricultural University, Nanjing, China; ^3^ University of Oxford, Oxford, United Kingdom; ^4^ China National Rice Research Institute, Chinese Academy of Agricultural Sciences, Hangzhou, China

**Keywords:** virulence, filamentous fungi, *Magnaporthe oryzae*, resistance, disease control

Filamentous fungi are a large group of various human and plant pathogenic fungi that seriously threaten people or plant health and cause huge economic losses. To successfully infect plants, some key regulators of fungi perform respective functions to ensure the formation of infectious fungi structures such as appressorium to interfere with plant immunity. Thus, exploring essential regulators for fungus virulence and the specific interaction mechanisms between fungi and plants is extremely important. This Research Topic aims to identify essential regulators that participate in the regulation of virulence of filamentous fungi, revealing their pathogenic mechanism and how they interact with plants. Meanwhile, we also present research on plant resistance to filamentous fungi and other related mechanisms of plant resistance changes.

Rice blast, caused by the filamentous fungi *Magnaporthe oryzae*, is one of the most serious threats to rice production worldwide (Pennisi, 2010; Du et al., 2022). Several essential regulators that participate in fungal virulence have been identified in recent years. These affect functional appressorium formation, reaction oxygen species, and so on (Yin et al., 2019; Li et al., 2020; Liu et al., 2020; Yin et al., 2020; Shi et al., 2021; Guo et al., 2023). Recently, the vitamin B6 synthase gene OsPDX1 has been found to play an essential role in rice stomatal immunity against pathogens (Liu et al., 2022). However, how the gene affects pathogens remains unknown. According to studies in our Research Topic, the pyridoxine biosynthesis protein MoPdx1 affects the appressorium function and pathogenicity of *M. oryzae* through vitamin biosynthesis. Deletion of *MoPDX1* leads to defects in the appressorium turgor pressure, which could be restored by exogenous vitamin B6, indicating that vitamins are involved in the development and pathogenicity of the fungus (Yang et al.), providing new insights into the roles of vitamins in fungal virulence.

Recently, beneficial microorganisms (including endophytic fungi) and their metabolism products have attracted significant attention as promising tools to control disease and improve crop yield, contributing to global food security (Cao et al., 2021; Lu et al., 2022; Wang et al., 2022). These also work in the prevention of rice blast. A study revealed that *Bacillus subtilis* KLBMPGC81 suppresses appressorium-mediated plant infection by altering the cell wall integrity signaling pathway and multiple cell biological processes in *M. oryzae*, providing new evidence of biocontrol of rice blast (Li et al.). In addition, another study reported diverse endophytic fungal species between grassy weeds with different herbicide resistances, suggesting that differences in endophytic fungi may contribute to the herbicide resistance of wheat fields in China (Zhan et al.).

Besides *M. oryzae*, researchers also systematically identified the Common in Fungal Extracellular Membrane (CFEM) domain containing proteins in another hemibiotrophic pathogenic fungus (*Marssonina brunnea*) in poplars (Qian et al.). This finding determined that four CFEM members are effectors of M. brunnea and provide valuable targets for further dissection of the molecular mechanisms underlying the poplar–*M. brunnea* interaction.

In summary, the studies in this Research Topic promote the understanding of fungi–plant interactions and indicate the immense potential of endophytic fungi on biological disease control.

## Author contributions

ZY and XD conceived and designed the topic, and also spread our topic. XL invited reviewers and controlled the quality of articles. YK and JH assisted in reviewing the manuscripts. All authors contributed to the article and approved the submitted version.
